# Epidemiological and Economic Evaluation of Alternative On-Farm Management Scenarios for Ovine Footrot in Switzerland

**DOI:** 10.3389/fvets.2017.00070

**Published:** 2017-05-16

**Authors:** Dana Zingg, Sandro Steinbach, Christian Kuhlgatz, Matthias Rediger, Gertraud Schüpbach-Regula, Matteo Aepli, Gry M. Grøneng, Salome Dürr

**Affiliations:** ^1^Veterinary Public Health Institute, University of Bern, Bern, Switzerland; ^2^Center of Economic Research, Swiss Federal Institute of Technology in Zurich, Zurich, Switzerland; ^3^Agricultural Economics, Swiss Federal Institute of Technology in Zurich, Institute for Environmental Decisions (IED), Zurich, Switzerland; ^4^Norwegian Veterinary Institute, Oslo, Norway; ^5^The Norwegian Institute of Public Health, Oslo, Norway

**Keywords:** decision-making, *Dichelobacter nodosus*, epidemiological modeling, economic effect, prevalence, ruminant, welfare, Switzerland

## Abstract

Footrot is a multifactorial infectious disease mostly affecting sheep, caused by the bacteria *Dichelobacter nodosus*. It causes painful feet lesions resulting in animal welfare issues, weight loss, and reduced wool production, which leads to a considerable economic burden in animal production. In Switzerland, the disease is endemic and mandatory coordinated control programs exist only in some parts of the country. This study aimed to compare two nationwide control strategies and a no intervention scenario with the current situation, and to quantify their net economic effect. This was done by sequential application of a maximum entropy model (MEM), epidemiological simulation, and calculation of net economic effect using the net present value method. Building upon data from a questionnaire, the MEM revealed a nationwide footrot prevalence of 40.2%. Regional prevalence values were used as inputs for the epidemiological model. Under the application of the nationwide coordinated control program without (scenario B) and with (scenario C) improved diagnostics [polymerase chain reaction (PCR) test], the Swiss-wide prevalence decreased within 10 years to 14 and 5%, respectively. Contrary, an increase to 48% prevalence was observed when terminating the current control strategies (scenario D). Management costs included labor and material costs. Management benefits included reduction of fattening time and improved animal welfare, which is valued by Swiss consumers and therefore reduces societal costs. The net economic effect of the alternative scenarios B and C was positive, the one of scenario D was negative and over a period of 17 years quantified at CHF 422.3, 538.3, and −172.3 million (1 CHF = 1.040 US$), respectively. This implies that a systematic Swiss-wide management program under the application of the PCR diagnostic test is the most recommendable strategy for a cost-effective control of footrot in Switzerland.

## Introduction

Footrot is an old disease in European countries, mentioned in France as early as the end of the eighteenth century (1). Early reports in Switzerland date to 1929 and 1965, indicating that the disease has been known for at least 100 years in this country (2, 3). Since then, the disease has spread to all regions of Switzerland, and is currently endemic (4, 5).

Footrot is an infectious disease, which mainly causes severe hoof lesions in sheep, but is also found in other ruminant species all over the world (4, 6–8). It is a multifactorial disease favored by humid environments with temperate climate. The main causative agent is *Dichelobacter nodosus*, although *Fusobacterium necrophorum*, aerobic diphtheroids, and coliforms are also reported to contribute to the development of clinical signs (9). The development and severity of disease depend on the climate, the virulence of the isolate, and the immune system of an individual animal (10, 11). Because the disease causes painful hoof lesions, it is not only of relevance for animal health but also for animal welfare. These painful lesions result in direct costs for the producers through weight loss and reduced wool production. In addition, consumers generally value animal welfare, so that there is a societal economic loss when animals are affected by footrot. In combination with the costs for treatment, the disease imposes a considerable economic burden in animal production (12). Management of footrot consists of regular hoof trimming, foot bathing, separation or elimination of affected sheep, and usage of antibiotics. These control measures are usually applied in combinations and are costly to the farmers. For example, a study in Great Britain estimated direct costs of £1.32 per ewe and £0.15 per lamb, summing up to costs of £24.4 for British producers annually (13). As control measures of single farmers cannot wipe out footrot, some countries implemented systematic programs to eradicate the disease. An economic study on a footrot eradication program in Western Australia found that the benefits of the program outweigh its costs at a ratio of 5.3:1 (14).

Footrot is not listed as a notifiable disease in the Swiss legislation. Nevertheless, all sheep farmers are obliged to comply with animal welfare legislations, which imply that clinically affected sheep has to be treated or slaughtered. In the cantons of Grisons (GR) and Glarus (GL), a coordinated management program was implemented in 1990 and 2013, respectively. The program consists of regular control of sheep herds, hoof trimming and foot bathing with formalin, zinc, or copper sulfate, and biosecurity measures. In case of footrot problems, these measures are executed more frequently, and infected animals are separated. The management program has been successful in reducing footrot prevalence within these cantons. Currently, policy is moving toward a nationwide coordinated control strategy against footrot in Switzerland, presuming that the disease will be listed as notifiable and controlled by law.

Epidemiological models are helpful and necessary tools to predict prevalence trends under different control strategies (15–17). Outputs of such models can be used for the economic evaluation of management strategies (18). Cost–benefit analyses of control strategies are important, and ideally conducted in an early phase of planning for potential control programs. Examples include highly infectious animal diseases such as foot-and-mouth disease (19) or classical swine fever (20, 21). Cost-effectiveness of control strategies for zoonoses such as rabies or brucellosis has also been studied, taking into account the costs for human deaths (22–24).

The objective of our study is to evaluate epidemiologic and economic aspects of different management strategies to reduce footrot prevalence in Switzerland. For this purpose, the direct costs of producers and the intangible costs of the society, mostly caused by affection of animal welfare, are considered. No distinction between the virulent and benign strain of *D. nodosus was made*. A cost–benefit analysis of four control strategies was conducted to inform policy makers who are considering an evidence-based nationwide coordinated control strategy of footrot in Switzerland.

## Materials and Methods

**Figure 1:**
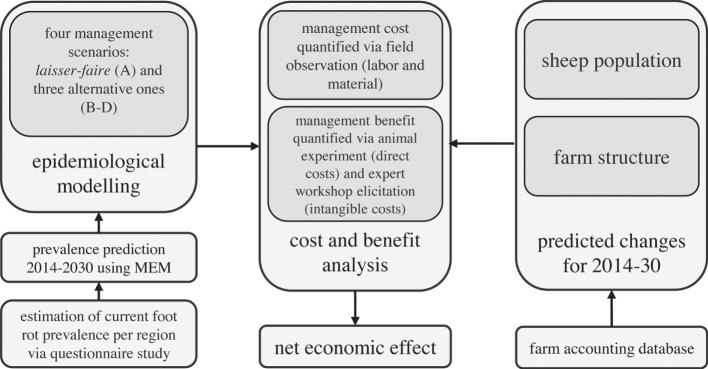
**Outline of the research project on the evaluation of the cost–benefit analysis of centrally organized control programs for footrot in the Swiss sheep population**.

The present study summarizes the results of a large project that evaluated the costs and benefits of centrally organized control programs for footrot in the Swiss sheep population. The entire project consisted of several successive subprojects (Figure 1). The animal experiment was approved by the Cantonal Veterinary Office of the Canton of Zug (approval number ZG 67/15) in accordance with the Swiss animal welfare legislation.

### Model Input Data

A questionnaire was sent to all sheep farmers of Switzerland aimed at revealing the current perceived prevalence of footrot in Swiss sheep premises (25). Questions on herd management, trade of animals, health issues involving claws, and management measures against footrot were also included. Of the 15,036 questionnaires sent out, 9,386 were returned, and 7,836 (52%) were usable for further analysis. Large premises contributed most to the questionnaire study, 79.6% of the total sheep population in Switzerland was covered by the completed questionnaires. Overall, 37% of the respondents stated that they experienced problems with footrot during the year 2014.

Estimates of the impact of footrot on sheep health were based on experimental controlled trial including a healthy versus footrot-infected sheep flock (25). Briefly, 85 lambs in the diseased group and 99 lambs in the control group were followed from birth to slaughter, which occurred at an individual weight of 42–46 kg. Reduction of the fattening period for healthy lambs was converted to economic benefit (see “Management Benefit”). The trial was also used to estimate labor costs, i.e., the time required for implementing of control measures on the farm.

### Definition of the Regions

A total of 19484 herds were integrated into the model. For conceptual reasons of the epidemiological model, Switzerland had to be divided into regions. These regions also served as basis for the regionalization for the maximum entropy model (MEM) and the cost–benefit analysis, considering varying costs and benefits between the different regions.

**Figure 2:**
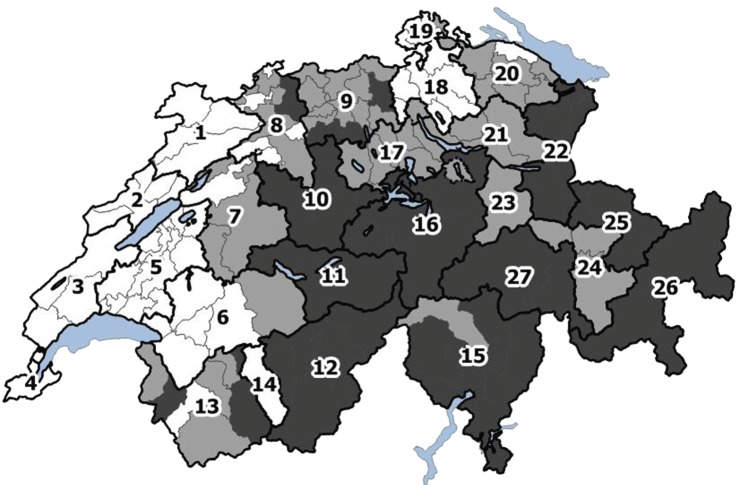
**Division of Switzerland into 27 regions according to sheep premises density, climate, and cantonal borders**. The colors reflect the tertiles of the density of sheep premises (number of sheep premises per square kilometer agricultural area): white: 0–0.54; light gray: 0.54–1.05; dark gray: 1.05–6.43.

Switzerland was divided into 27 regions for the footrot model (Figure 2). Two criteria were used for the allocation of the regions: density of sheep premises (first criterion) and the climate (second criterion). Data to inform the sheep premises density were sourced from the AGIS database (agrarian policy information system of Switzerland) and data were calculated as the number of premises per agricultural area per political district. The AGIS database only records data on professional premises and therefore non-professional premises were not considered for the classification of densities. District densities were divided into three categories using tertiles as limits. The transmission of footrot is also influenced by the climate in which mainly temperature and precipitation are seen as relevant factors (26, 27). Switzerland is divided into 12 climatic regions. Following these climatic regions, the density-classified regions were further subdivided or merged. In a final step, large regions with the same density and climate were subdivided following cantonal borders to avoid large differences in size between regions. For each region, the population size (number of sheep premises according to the AGIS database) and a climatic factor were calculated (Appendix in Supplementary Material). Currently, a footrot control program is mandatory for all sheep premises and implemented in the regions 23–27 (situated in the cantons of GR and GL).

### Estimation of Current Prevalence of Footrot Using MEM

To account for the non-respondents of the questionnaire study and to extrapolate the prevalence estimates per region to entire Switzerland, an MEM was used (25). The MEM is a Bayesian method that integrates *a priori* information to estimate the probability of the occurrence of an unknown variable (28). Here, the maximum likelihood estimator was used to estimate the probability of footrot prevalence in the defined regions. To ensure stability of the MEM, regions with <200 herds had to be complied, leading to 22 regions out of the 27 regions (regions 1 and 2 were compiled, as well as regions 3 and 4, 13 and 14, 18 and 19, and 23 and 24).

*A priori* information included geographic location (region), farm size (number of animals, growth rate, and agricultural area), structural features of farms (whether or not the farm holds rams or keeps animals on pasture, age of the farmer), and contact information (exhibitions and pasturing) are used, sourced from the questionnaire and AGIS database. This *a priori* information was combined with the prevalence of farms per region that stated to have experienced problems with footrot in 2014. The model was tested by predicting footrot status of the farms within the sample where the status was known. The econometric model had a fit above 70% (measured as pseudo-*R*^2^), implying that the model mimics the data-generating process well. Neither selection nor information bias was expected. It was then applied to the entire Swiss sheep farm population to estimate the footrot prevalence within each region (Table S1 in Supplementary Material). This prevalence was further used as a starting point for the epidemiological model. Within each of the compiled regions, the same prevalence was used (Table S1 in Supplementary Material).

### Epidemiological Model

#### Model Structure

**Figure 3:**
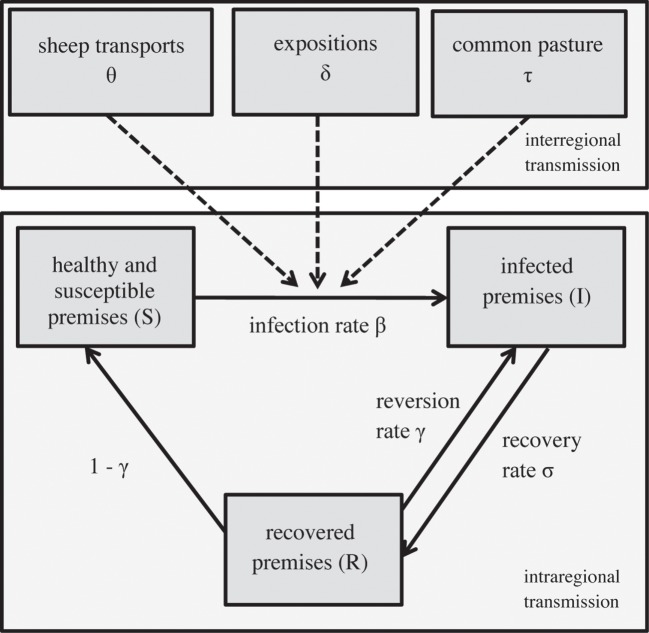
**Structure of the epidemiological model simulating within and between the regional spread of footrot, modified from (29)**.

The footrot transmission model has been developed based on a stochastic susceptible-infected-recovered compartmental model designed to simulate a footrot outbreak in Norway (29). The model was implemented in *R*^1^. The model allows the simulation of the spread of the disease within and between defined geographical regions, using the sheep premises as the smallest unit (Figure 3). The time step of the simulation is 1 year.

#### Simulation of the Spread within a Region

Premises were grouped into three compartments within a region: susceptible (*S*), infected (*I*), and recovered (*R*) premises (Figure 3). Susceptible premises get infected with an infection rate β and recover afterward with a recovery rate σ. Subsequently, they either become re-infected (with the reversion rate γ) or again susceptible with a rate of 1 − γ. The spread between the compartments within a region *i* at the time *t* is:

(1) $$S_{i,t+1}=S_{i,t}+\left(1-\gamma_{i}\right)*R_{i,t}-\beta_{i\;}*\;S_{i,t}\;*\;I_{i,t}$$

(2) $$I_{i,t+1}=I_{i,t}-\;\sigma_{i}*I_{i,t}+\beta_{i}*S_{i,t}*I_{i,t}+\gamma_{i}*R_{i,t}$$

(3) $$R_{i,t+1}=R_{i,t}+\sigma_{i}*I_{i,t}-\gamma_{i}*R_{i,t}-\left(1-\gamma_{i}\right)*R_{i,t}$$

The population size *N* = *S* + *I* + *R* per region *i* was sourced from the AGIS database. The regional prevalence at the start of the simulation was informed by the output of the MEM (Table S1 in Supplementary Material). The infection rate β is a stochastic parameter (pert-distribution) calculated separately for each region and incorporates the regional sheep premises density and the climate (Appendix in Supplementary Material).

The recovery and the reversion rates were incorporated as stochastic parameters (uniform distributions, Appendix in Supplementary Material) with separate values for regions with and without mandatory footrot control programs. Depending on the scenarios simulated, the regions with and without mandatory control program are varying.

#### Simulation of the Spread between the Regions

Spread of footrot between regions is implemented in three ways: sheep transport (trade), common pasture, and sheep expositions. Sheep transports are possible across entire Switzerland. The number of newly infected premises per year via this transmission pathway (θ*_j,i_*) was calculated out of the annual number of sheep transports on herd level from regions *j* to *i*, the proportion of infected premises in the sending region *j* and the proportion of susceptible premises in the receiving region *i* (Appendix in Supplementary Material). Sheep movement data were sourced from the questionnaire study in which each farmer was asked to state the two cantons—apart from the home canton—where the majority of sheep has been sent to and received from in the last 12 months.

The transmission from region *j* to *i* via common pasture (parameter τ*_j,i_*) and interregional sheep exhibitions (parameter δ*_j,i_*) follow the same principle. Animals of different regions come together, get infected with the transmission rate β_pasture_ and β_Expo_, respectively, and go back to their premises at the end of the summer or exhibition, where they may infect other animals and premises. The number of newly infected premises per year via common pasture (τ*_j,i_*) was computed using information on the number of sheep herds from both regions *i* and *j* that spend the summer on common pasture, herd density and climate on the pastures, the proportion of infected herds in region *j*, and the proportion of susceptible herds in region *i* (Appendix in Supplementary Material). The number of sheep sent to common pasture for each region was sourced from the questionnaire. The size of common pasture area was sourced from the AGIS database, which was required to calculate the herd densities (herds per square kilometer) on pastures. Similarly, the number of newly infected premises per year via exhibitions (δ*_j,i_*) was calculated out of the number of sheep herds exhibited per year and regions *i* and *j*, the herd density and climate on the site of exposition, the proportion of infected herds in region *j*, and the proportion of susceptible herds in region *i* (Appendix in Supplementary Material). Information on the number of sheep herds exhibited by each region for the large interregional expositions was provided by the Swiss Sheep Breeding Association^2^.

As a result of the integration of the spread between the regions, subsuper1 1–3 were expanded so that the number of premises in each compartment of region *i* and year *t* is calculated as follows:

(4) $$\begin{array}{l} S_{i,t+1}=S_{i,t}+\left(1-\gamma \right)*R_{i,t}-\text{min }\left[\left(\beta_{i}*S_{i,t}*I_{i,t}+\sum_{j\neq i}\;\theta_{j,i,t}\right.\right.\left.\left.+\sum_{j\neq i}\;\tau_{j,i,t}+\sum_{j\neq i}\;\delta_{j,i,t}\right),S_{i,t}\right] \end{array}$$

(5) $$\begin{array}{l} I_{i,t+1}=I_{i,t}-\sigma *I_{i,t}+\text{min }\left[\;\left(\;\beta_{i}*S_{i,t}*I_{i,t}+\sum_{j\neq i}\theta_{j,i,t}+\sum_{j\neq i}\tau_{j,i,t}\right.\right.\left.\left.+\sum_{j\neq i}\delta_{j,i,t}\right),S_{i,t}\right]+\gamma_{i}*R_{i,t} \end{array}$$

(6) $$R_{i,t+1}=R_{i,t}+\sigma *I_{i,t}-\gamma_{i}*R_{i,t}-\left(1-\gamma_{i}\right)*R_{i,t}$$

where *i* and *j* denote the region receiving and transmitting footrot.

#### Global Sensitivity (GSA) Analysis

A GSA analysis was applied that differs from the classical “one-parameter-at-a-time” SA by considering interactions between the parameters (30). In total, 13 parameters were included within the GSA. These include the number of susceptible (*S_i,t_*_=1_) and infected (*I_i,t_*_=1_) herds per region *i* at the start of the simulation (*t* = 1), the three interregional parameters (infection rate β*_i_*, recovery rate σ*_i_*, and reversion rate γ*_i_*) per region, the number of sheep herd transports between region *i* and *j* (MSh*_j,i_*), the number of sheep herds sent to common pastures (*n*_pasture_,*_i_*), and exhibitions (*n*_Expo_,*_i_*), respectively, and the herd density and climate on common pastures and exhibitions, respectively (*d*_pasture_, Cl_pasture_, *d*_Expo_, Cl_Expo_). In addition, the mean of all infection rates β*_i_* was incorporated, which was used to calculate the infection rates on pastures and exhibitions. For the GSA, all parameters were allowed to vary between ±10% around their original value. The function “soboljansen” from the *R* package “sensitivity” was used (31, 32). One hundred and fifty thousand iterations were needed to result in narrow enough confidence intervals of the Sobol indices, the measures of the parameters’ influence on the footrot prevalence.

### Fitting of the Model to the Swiss Situation

To fit the model to the Swiss situation, it was assumed that footrot is currently in a stable endemic stage in Switzerland, thus the prevalence per region is constant over time. This assumption was made based on evidence of existence of the disease in the surrounding countries (Germany and France) since at least end of the eighteenth century (1, 3) and on a study providing evidence than footrot exists in all regions of Switzerland (5). The parameter values of β, σ, θ, τ, and δ were calculated and incorporated in the model as described above and in the Appendix in Supplementary Material. The value of the reversion rate γ was fitted to the countrywide prevalence in Switzerland so that the model output came as close as possible to the target prevalence of 40.2%, estimated by the MEM. Reversion rate values of 40–55% were tested with steps of 1%. The value of γ for regions 23–27 (cantons of GR and GL) was defined to be smaller than the one for the other regions, based on the ratio of the reversion rates calculated from the questionnaire dataset (43.6% for premises undergone a footrot control program on herd level, 74.5% for those that did not undergo such a program).

Because the Swiss-wide prevalence was used as the measure to fit the model and prevalence in the different regions deviated from the start value estimated by the MEM over the course of the simulated years (running time of the model = 100 years), a correction algorithm had to be applied. For each region *i*, a correction factor *k_i_* was calculated based on the target prevalence (target_prev*_i_*, MEM outcome) and prevalence estimated by the simulation model at year 45 (prev*_t_* = _45_,*_i_*, year with prevalence closest to the target value, see “Fitting to the Swiss Situation and Calculation of Reversion Rate γ”), so that

(7) $$k_{i}=\frac{\text{target\_pre}{\text{v}}_{i}}{\text{pre}{\text{v}}_{t=45,i}}.$$

### Description of the Scenarios

Four scenarios were defined (Table 1). For each scenario, 1,000 simulations were conducted and the mean, median, and 2.5 and 97.5‰ of the footrot prevalence were extracted for results presentation and further analysis. Each simulation ran over 100 years and started with the parameter values described above.

**Table 1:** **Definition of scenario with their recovery and reversion rate values for regions 1–22 (no mandatory footrot program implemented) and regions 23–27 (mandatory footrot program implemented)**.

Scenario	Values of the parameter (recovery and reversion rate)	
	Regions 1–22	Regions 23–27 (canton GR and GL)
A (*laisser-faire*): current control strategies ongoing with mandatory control program with polymerase chain reaction (PCR) diagnosis in regions 23–27 only	Recovery rate: uniform (20.0–24.5%; mean 22.3%)	Recovery rate: uniform (41.1–50.2%; mean 45.6%)
	Reversion rate: uniform (44.1–53.9%, mean 49.0%)	Reversion rate: uniform (8.6–10.5%; mean 9.5%)
B: nationwide mandatory control program without PCR diagnosis	Recovery rate: uniform (41.1–50.2%; mean 45.6%)	
	Reversion rate: uniform (33.1–40.5%; mean 36.8%)	
C: nationwide mandatory control program with PCR diagnosis	Recovery rate: uniform (41.1–50.2%; mean 45.6%)	
	Reversion rate: uniform (8.6–10.5%; mean 9.5%)	
D: all footrot control measures ceased in Switzerland	Recovery rate: uniform (20.0–24.5%; mean 22.3%)	
	Reversion rate: uniform (56.6–69.1%; mean 62.8%)	

Scenario A (*laisser-faire*) was defined as the current status of footrot control in Switzerland and used as the baseline when different scenarios were compared. Recovery and reversion rates differed between regions 23 and 27 (located in canton GR and GL, mandatory control program ongoing) and regions 1–22 (rest of Switzerland, no mandatory control program implemented). It was assumed that in the future, the newly developed polymerase chain reaction (PCR) diagnostic test (33) will be considered in the regions with mandatory control program. This test also detects non-clinical animals, which results in a higher sensitivity of the footrot detection and in consequence in a lower reversion rate (Appendix in Supplementary Material).

Scenarios B and C were defined as extension of the mandatory control programs as currently implemented in the cantons of GR and GL to a nationwide level including all regions. This program consists of separation of the infected herd, hoof trimming, and regular foot bathing^3^. In scenario B, no PCR diagnostic test was considered and the definition of a premise being footrot free was based on clinical signs only, where every single sheep was tested. In scenario C, PCR was considered for the detection of footrot, addressing a given proportion of sheep (ranging from 100% for small herds to 10–40% for large herds). Examination by a veterinary (scenario B) or a PCR test (scenario C) and a hoof inspector are conducted in the first year of the sanitation.

For scenario D, it was assumed that all mandatory control measures were ceased in Switzerland. This comparison is relevant because the current benefit of existing management strategies should be assessed. The recovery rate was estimated based on the questionnaire database for premises that did not undergo a footrot control program. The reversion rate γ*_D_* was calculated from the fitted reversion rate γ (49.0%, see “Fitting to the Swiss Situation and Calculation of Reversion Rate γ”) and ratio between the reversion rate of premises with no herd level control measures applied (γ_non-controlled premises_, 74.5%) and the reversion rate calculated from the entire questionnaire dataset (γ_entrie_dataset_, 58.1%):

(8) $$\gamma_{D}=\frac{\gamma }{\gamma_{\text{entrie\_dataset}}}*\gamma_{\text{non-controlled premises}}.$$

After the model simulation, the model output of all scenarios per region *i* and year *t* was corrected by the correction factor *k_i_*. For each scenario, the final regional prevalence in the year *t* was calculated at:

(9) $$\text{prev\_fina}{\text{l}}_{t,i}=\text{prev\_modelOutpu}{\text{t}}_{t,i}*k_{i}.$$

### Cost–Benefit Analysis

The costs and benefits were calculated for each scenario according to how many herds were infected, susceptible, and recovered in each year. The cost–benefit analysis is a systematic approach for evaluating the economic implications of management scenarios. The aim of this analysis is to identify the management strategy maximizing the net welfare effect, which is we call net economic effect to avoid confusion with animal welfare. This method is frequently used to evaluate policies that aim at an improvement of animal health. To quantify the economic implications of footrot management, the net economic effect was measured with the net present value method as follows:

(10) $$\text{NPV}\left(d,T\right)=\sum_{t=1}^{T}\;\frac{\left(\sum_{j=1}^{J}b_{j,t}-\sum_{i=1}^{I}c_{i,t}\right)}{{\left(1+d\right)}^{t}}-\sum_{i=1}^{I}\;c_{i,0}$$

where the year was denoted with *t*, the discount rate with *d*, the benefits of management with *b*, and the costs of management with *c*. The costs and benefits consist of a number of components, which are summarized by *i* and *j*. The net economic effect was calculated at the farm level and then aggregated at the nation level.^4^ The benefits of improved animal welfare were also considered in our analysis. However, as these benefits are not direct farm benefits, they were only considered at the national level. The cost–benefit analysis is concerned with the period 2014–2030. The analysis was limited to this period because uncertainty increases over time. For the evaluation of the effect of disease control, the time period after the implementation is of largest interest. The discount rate was assumed to be 1 during calculation period because the inflation rate in Switzerland remained close to 0 in the last years, although there is considerable uncertainty with respect to future economic development. Similarly, it was assumed that prices and salaries will remain at their respective level in 2014. The implementation of footrot measures affects the supply of Swiss sheep products and, therefore, their market prices. Price changes affect rents on the consumer and producer side [Ebel et al. (34)]. Such indirect economic effects of footrot are not taken into account in the conducted cost–benefit analysis, but are discussed below. Because the Swiss sheep industry has undergone major changes in recent years, it was necessary to predict the future sheep population and farms structure before assessing costs and benefits of the management of footrot.

#### Predicting the Future Sheep Population

The size of sheep population for 2014–2030 was estimated with historical data on sheep farming in Switzerland from the farm accounting database (AGIS database). This database contains information on the entire sheep population in Switzerland for 1999–2014 (Table S2 in Supplementary Material). The size of the sheep population in each region was calculated for every year. The data show that the number of sheep has been increasing over this period. However, the development is not homogenous with some regions observing a substantial decrease in the sheep population (regions 26 and 27) and others a substantial increase (regions 1 + 2, 9, and 17). Considering the substantial variation in the development of the sheep population, it is necessary to apply an identification strategy for the future sheep population that accounts for this heterogeneity. A number of regression specifications were compared to obtain a correct identification of the relationship using the farming data for 1999–2014.^5^ It was found that the seemingly unrelated regression model with region-specific fixed effects and linear time trends replicates the data-generating process most appropriately. The regression model was developed by Zellner (35) and allows correlation in the error terms. The equation system is outlined below:

(11) $$S_{i,t}=\alpha_{i}+\beta_{i}T_{i,t}+\varepsilon_{i,t},\;E\left[\varepsilon_{i,t}*\varepsilon_{k,t}|T_{t}\right]=\sigma_{i,k}$$

where *i* represents the equation number (region) and *t* the year. The region fixed effects were denoted with α*_i_* and the region-specific linear time trend with *T_i,t_*. The error term was denoted by ε*_i,t_*, which was allowed to be correlated across regions but not over time. The system of equations was solved simultaneously using the feasible general least squares method. The estimation results are summarized in Table S3 in Supplementary Material and illustrated in the Figure S1 in Supplementary Material. Most regions showed a highly significant and positive trend in the sheep population, and the largest effects are found in regions 7, 9, 10, and 20. The regression specifications fitted the underlying data well, which is indicated by the generally high predictive power (*R*^2^ values).

#### Predicting the Future Farm Structure

The management costs were expected to vary between farm types. Larger farms were expected to benefit from scale effects because they can use their equipment more efficiently. Hence, the average fixed and variable cost of treatment per unit was expected to be substantially lower for larger farms. To account for scale effects (reduction in average cost per unit of output by increasing the production), sheep farmers were classified in each region according to the scale of their operation as small (1–30), medium (31–70), and large operations (>70). Substantial differences could be observed in the farm size between regions (Table S4 in Supplementary Material). Although most farms in Switzerland were classified as small operations, this share has been decreasing substantially since 1998. Therefore, sheep farming activities in Switzerland are becoming more professional with mostly small farms ceasing and large farm expanding their activities. To model future scale effects, the same regression model as used for the prediction of the sheep population was applied. The regression results are presented in Table S5 in Supplementary Material. It was found that the proportion of small and medium operations will decrease further in the future. Particularly for the southern and alpine part of Switzerland (regions 13–15 and 25–27), an increase in the size of farms is expected.

#### Management Cost

The management cost by farm type was defined according to the four management scenarios. They consist of labor costs on the farm, third-party labor costs, and material costs. In regions with mandatory control, the cost items consisted of hoof trimming and weekly hoof bathing over a period of 10 weeks for infected farms, four control visits in the first year and one each in the two consecutive years. In scenario B, control visits on farms include clinical inspection of all animals. In the other scenarios, samples for the PCR diagnostic test were taken during the control visits to identify infected animals. The PCR test is assumed to be conducted by trained personnel and only a proportion of animals were tested per herd (ranging from 100% for small herds to 10–40% for large herds), prioritizing high risk animals (lame animals, newly purchased animals, rams, and heavy ewes). This implies substantially lower management cost, which is accounted for as third-party labor costs. On the other hand, the additional laboratory cost of the PCR test increased the material costs (CHF 6.50 per test). For regions without control program, management activities were reduced to the minimal level defined by the animal welfare legislation. Costs related to this included hoof trimming and hoof spray. A detailed summary of the management approach and the management cost for the different scenarios is provided by Aepli et al. (25).

#### Management Benefit

The management benefit is composed of farm benefits and the reduction of intangible damage. Farm benefits arise mainly from reduction in fattening time. It was found in the experimental animal trial that the fattening time was significantly longer for infected lambs than for non-infected lambs (31.9 days longer, *p* < 0.01, linear mixed model) (25). An additional day of fattening was valued with CHF 2.70 (1 CHF = 0.918 € or 1.040 UD$), which is composed of feed cost (CHF 0.15), operation cost (CHF 0.25), and labor opportunity cost (CHF 2.30).

Intangible costs are not directly quantifiable costs that are related to an identifiable source. Therefore, they can be seen as external costs, which are not taken into account in the cost calculation of the producers. These costs were measured with the help of a structured expert elicitation. Two workshops were conducted in which stakeholders such as farmers, consumers, veterinaries, scientists, and government employees discussed the intangible costs of footrot. It was found that intangible costs are primarily related to the negative utility of society due to reduced animal health and limitation of natural behavior. As an average of the two workshops, the experts concluded that these two animal welfare issues contribute 84% of intangible costs. The monetary value of pain caused by footrot was then estimated using a similar method as proposed by Fitzpatrick et al. (36). Based on the discussed intangible cost components and the evaluated societal valuation of animal pain, the experts estimated the national costs of footrot. While there was a wide variation in the single expert opinions on the society values animal welfare, the workshop participants generally agreed with the mean monetary value derived in the workshop. The experts concluded that the annual nationwide intangible cost caused by footrot with a national prevalence of 70% equals CHF 53.03 million. The cost at prevalence rates of 0, 20, and 50% was evaluated as well. Piecewise cubic Hermite interpolation was used in succession to calculate the intangible cost for each prevalence level, in 0.1% steps. A more detailed description of the elicitation approach and results is provided by Aepli et al. (25).

## Results

### Fitting to the Swiss Situation and Calculation of Reversion Rate γ

For the fitting procedure, the model started with a prevalence of 40.5%. This value was closest to the prevalence of 40.2% (target prevalence) while avoiding partial herds. At year 45, the model reached the target prevalence and stayed in an endemic steady-state afterward (variation of 40.38–40.48%; Table 2; Figure S2 in Supplementary Material). Year 45 was therefore defined as the year of data collection (year 2014) and the year when the alternative strategies where implemented (Figure S3 in Supplementary Material).

**Table 2:** **Footrot prevalence in % during the fitting process to the Swiss situation up to simulation year 45, which was defined as year 2014**.

	Year 2	Year 5	Year 10	Year 15	Year 45
Median	38.33	37.97	38.95	39.56	40.38
Mean	38.33	37.97	38.95	39.55	40.35
2.5‰	37.19	35.33	34.7	34.31	33.73
97.5‰	39.51	40.59	43.09	44.65	46.67

*Statistics of 1,000 simulations*.

The value of the reversion rate γ, which resulted in a model prevalence closest to the target prevalence, was determined at 49.0% for the regions 1–22 and 36.8% for regions 23–27.

### Footrot Prevalence under Scenarios A–D

**Figure 4:**
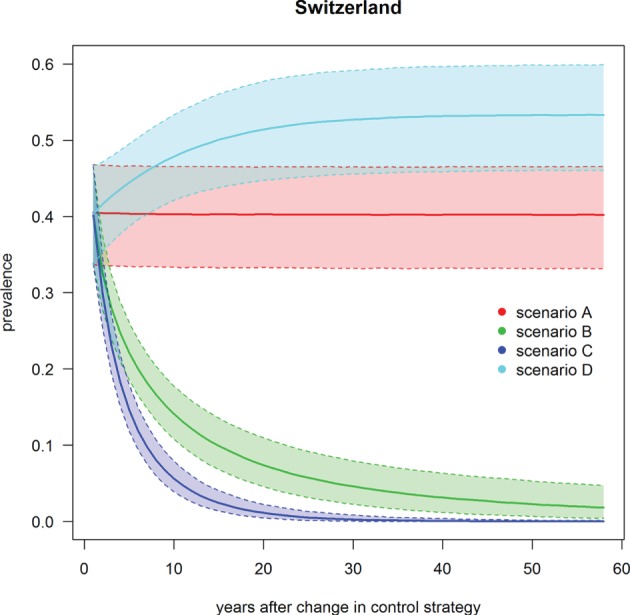
**Trend of footrot prevalence for the four different scenarios A–D for entire Switzerland**. Lower dashed line = 2.5‰, upper dashed line = 97.5‰, solid line = median out of 1,000 simulations.

**Figure 5:**
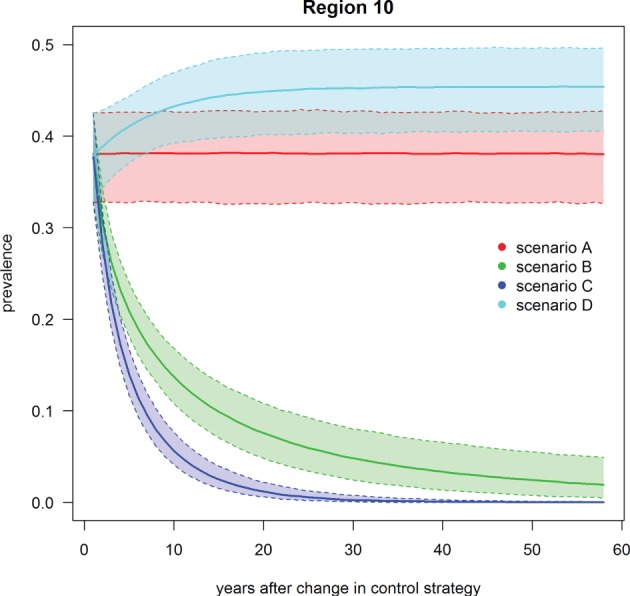
**Trend of footrot prevalence for the four different scenarios A–D for region 10 (example for a region without mandatory control program)**. Lower dashed line = 2.5‰, upper dashed line = 97.5‰, solid line = median out of 1,000 simulations.

**Figure 6:**
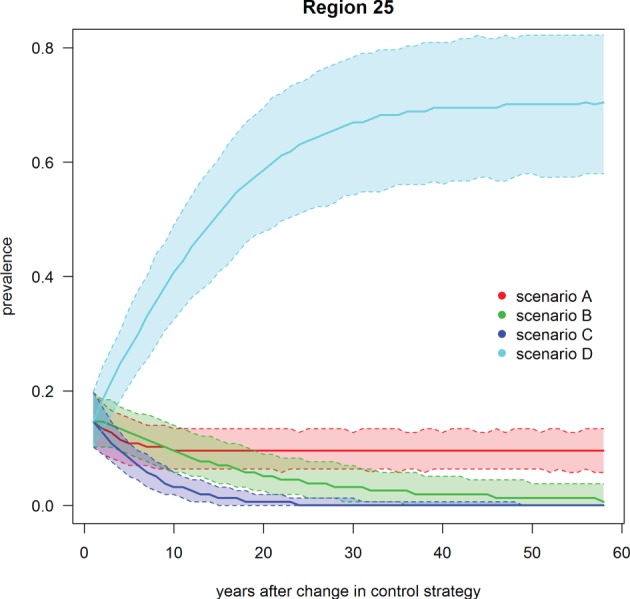
**Trend of footrot prevalence for the four different scenarios A–D for region 25 (example for a region with mandatory control program)**. Lower dashed line = 2.5‰, upper dashed line = 97.5‰, solid line = median out of 1,000 simulations.

Scenario A was defined as the current state of footrot control, i.e., mandatory control program in regions 23–27 only, however, with the introduction of a new PCR diagnostic test in these regions. The nationwide prevalence and the prevalence in regions without mandatory control program only decreased slightly (<1%) over time (Table 3; Figures 4 and 5). For the regions with mandatory control program, a decrease in the prevalence was observed because of improved disease detection and consequently lower reinfection of controlled premises (Figure 6). On average these regions had a median prevalence of 25.5% at the beginning of the simulations. After 18 years of simulation, a plateau was reached at a median prevalence of 18% for the regions 23–27.

**Table 3:** **Prevalence (%) of footrot of the scenarios A–D 2, 5, 10, 15, and 20 years after implementation (scenarios B and C) or cease (scenario D) of the respective control measurements**.

Years after scenarioimplementation		A	B	C	D
2	Median	40.41	27.99	23.08	42.65
	Mean	40.27	28.02	23.11	42.51
	CI 2.5%	33.53	23.97	19.44	36.65
	CI 97.5%	46.67	32.32	27.08	47.88
5	Median	40.33	19.97	11.87	45.26
	Mean	40.18	20.01	11.96	45.17
	CI 2.5%	33.40	16.50	9.38	39.59
	CI 97.5%	46.63	23.81	14.95	50.34
10	Median	40.26	13.06	4.74	48.38
	Mean	40.13	13.12	4.81	48.32
	CI 2.5%	33.31	9.79	3.16	42.58
	CI 97.5%	46.54	16.74	6.86	53.95
15	Median	40.28	9.32	2.09	50.38
	Mean	40.10	9.39	2.17	50.30
	CI 2.5%	33.31	6.27	1.12	44.03
	CI 97.5%	46.53	12.89	3.60	56.42
20	Median	40.29	7.01	0.97	51.62
	Mean	40.10	7.12	1.04	51.51
	CI 2.5%	33.28	4.23	0.38	44.85
	CI 97.5%	46.54	10.54	2.02	57.97

*Thousand simulations were conducted per scenario*.

Scenario B was defined as the introduction of Swiss-wide mandatory control measures as currently implemented in the cantons of GR and GL, without using the PCR diagnostic test (only clinical diagnosis considered). A clear decrease in the nationwide prevalence was observed during the first year of simulation (Table 3; Figure 4). In the first 2 years of simulation, the median of the Swiss prevalence decreased from 40.4 to 28.0% (mean 28.0%, 95% CI 24.0–32.3%). The 10% mark was reached at year 14 with a median prevalence of 10.0% (mean 10.0%, CI 6.8–11.5%). In the following years, the prevalence further decreased continuously to a value of 1.8% (mean 2.0%, CI 0.4–4.7%) at the end of the simulation (year 57). Elimination of footrot (median prevalence of 0%) was only reached in regions 4 and 14 after 42 and 28 years of simulation, respectively. On average, the prevalence in the regions 1–22 fell more rapidly than that of the regions 23–27 (Figures 5 and 6). The prevalence of 10% was reached after a mean of 13.5 years (6–28 years for the different regions) and after a mean of 21 years (9–30 years) for the regions 1–22 and 23–27, respectively.

Scenario C was defined as the introduction of Swiss-wide mandatory control measures as currently implemented in the cantons of GR and GL, but including the use of the PCR diagnostic test. The effect of the PCR diagnostics can therefore be observed by comparing scenario C with B. Shortly after the implementation of the control measures, the prevalence decreases even more rapidly than in scenario B. Starting at a nationwide median of 40.4%, it fell to 23.1% (mean 23.1%, CI 19.4–27.1%) after 2 years (Table 3; Figure 4). In the following years, the prevalence rapidly decreased further so that after 6 years of simulation the median prevalence fell below 10% and after 20 years to 1.0% (mean 1.0%, CI 0.3–2.0%). After 50 years of simulation, footrot is predicted to be eliminated on average (median nationwide prevalence of 0%). Only slight differences were observed between the regions 1–22 and 23–27 (Figures 5 and 6). The 10% prevalence was reached earlier for the median of the regions 1–22 (after 6 compared to after 7 years) and the footrot elimination (0% median prevalence) was achieved earlier for the regions 23–27 (after 24 compared to after 33 years).

Scenario D was defined as the cease of all mandatory control measures in Switzerland. The median of the Swiss prevalence increased slightly in the first 2 years up to 42.7% (mean 42.5%, CI 36.7–47.9%) (Table 3; Figure 4). An increase of 10% to a median of 50.4% (mean 50.3%, CI 44.0–56.4%) was observed after 15 years of simulation. This increasing trend continued and toward the end of the simulation (year 57), the median of the prevalence reached a plateau, which was 13% higher than at the beginning of the simulation (median 53.3%, mean 53.2%, CI 46.0–59.9%). The increase in median prevalence was faster in the regions 23–27, where the cease of the mandatory control program had a direct effect (Figure 6), than for the regions without earlier implemented control programs (Figure 5).

### GSA Analysis

Two parameters were detected to mostly influence footrot prevalence (the outcome of the model). These are the recovery rate σ and reversion rate γ with total effect Sobol indices of 0.69 and 0.61, respectively. To a lower extent, infection rate β (total effect Sobol index = 0.49) and the number of susceptible herds at the beginning of the simulation (total effect Sobol index = 0.48) also resulted in Sobol indices slightly higher than for the other parameters, which range from 0.43 to 0.45 (Figure S4 in Supplementary Material). The total effect Sobol index also integrated the interactions between the respective parameter and with all other parameters tested in the GSA.

### Cost and Benefit Evaluation

Table 4 summarizes the cost and benefit of footrot management under the four management scenarios for 2014–2030. Among the components of management cost, labor cost accounted for the largest share in total cost. The smallest management costs were found under scenario C, and the highest costs were expected with scenario D. In comparison to scenario C, labor costs under scenario B were substantially larger. This is because PCR tests are less labor demanding than footrot inspections and this includes both, on-farm labor as well as third-party labor. Most of the total management costs of scenario C occur in the initial years after the management strategy was implemented, and cost decreases substantially in the following years as the prevalence rate drops. For the benefits, it was found that under scenario D, the fattening time would increase substantially and the animal welfare would decrease. By increasing the management intensity (scenarios B and C), a substantial decrease in fattening time and improvement of animal welfare could be achieved. The effect was larger for scenario C, where the benefit for reduced fattening time increased to CHF 52.8 million. While the intangible cost in scenario C is still nearly double as high as the gain through reduced fattening time, its value of 99.9 Mio. CHF is substantially lower than in any other scenario.

**Table 4:** **Cost and benefit of footrot management for 2014–2030 (in 1,000 CHF)**.

Scenario	A	B	C	D
**Management costs**				
On-farm labor	47′135	14′244	8′080	59′477
	(38′707–55′203)	(11′180–17′611)	(6′205–10′297)	(51′648–66′793)
Third-party labor	985	12′451	7′922	0
	(669–1′339)	(10′265–14′851)	(6′601–9′484)	(0–0)
Material cost	2′020	763	414	2′591
	(1′656–2′370)	(588–956)	(311–536)	(2′248–2′910)
**Total costs**	50′140	27′458	16′416	62′068
	(41′031–58′912)	(22′034–33′418)	(13′117–20′317)	(53′896–69′703)
**Economic consequences for footrot development**				
Benefit from shorter fattening time	1′648	40′665	52′754	−20′474
	(1′328–1′802)	(36′921–43′821)	(45′788–59′249)	(−20′901 to −19′540)
Intangible cost (animal welfare and others)	553′422	192′775	99′936	691′667
	(441′966–647′156)	(140′771–253′578)	(71′947–134′365)	(606′668–762′332)

*The discount factor is 1. All cost and benefit are expressed in constant 2014 prices. Direct cost and treatment cost are summed over time of the management period (2014–2030). The 95% confidence interval is reported in parenthesis. The total net economic effect is presented in Table 5*.

*Cost differences between small, medium, and large farms are taken into account. Composition of the cost categories depends on the scenario. On-farm labor costs are calculated as 28 CHF/h times the farm personnel’s time estimated for foot bathing, hoof trimming, and presence at clinical inspections or collection of samples for diagnostic tests. Third-party costs include clinical inspections by hoof controllers and veterinaries and diagnostic tests. Material costs include water and zinc sulfate. The saved costs associated with the reduction of fattening time were calculated by assuming that the costs per animal are 2.70 CHF/day, and animals are not prematurely slaughtered. Intangible costs are calculated based on national prevalence rates, given the results of the expert elicitations*.

**Table 5:** **Net economic effect of scenario B–D compared to scenario A (*laisser-faire*) in 1,000 CHF**.

Scenario	B	C	D
**Management costs (compared to scenario A)**			
Difference in labor cost	−32′891	−39′055	12′342
Difference in third-party labor cost	11′466	6′937	−985
Difference in material cost	−1′257	−1′606	571
**Total of cost differences**	−22′682	−33′724	11′928
**Management benefits (compared to scenario A)**			
Difference in direct benefits (reduced fattening time)	39′017	51′106	−22′122
Reduction in intangible cost (animal welfare and others)	360′647	453′486	−138′245
**Total of benefit differences**	399′664	504′592	−160′367
**Cost–benefit**			
Direct net economic effect	61′699	84′830	−34′050
Net economic effect, direct and intangible	422′346	538′316	−172′295

*The total of cost and benefit are reported for the period 2014–2030. All cost and benefit are expressed in constant 2014 prices*.

The net economic effect of footrot management was calculated by comparing the alternative management scenarios B–D with the baseline scenario A (Table 5). It was found that under scenario D, the management cost will increase by CHF 11.9 million. Since the management benefit will also be reduced by CHF 160.4 million, the net economic effect of scenario D is negative (CHF −172.3 million), indicating that it is less preferable than the laissez-faire scenario A and clearly the least preferable option among the compared scenarios. In contrast, scenarios B and C have a positive net economic effect of CHF 422.3 and 538.3 million, respectively. In both scenarios, reductions of intangible costs are the largest fraction of economic gains. Given that the sanitation measures have to be paid by the farmers and intangible cost reductions are social gains, it is worthwhile to note that there is also a positive benefit due to shortened fattening time—which is a benefit received directly by the farmer. It was found that the management costs were substantially lower for scenario C than for scenario B. Moreover, due to higher accuracy of the PCR method in recognizing footrot, management benefits were estimated to be larger for scenario C than for scenario B.

## Discussion

The aim of this study was to evaluate the current footrot situation in the Swiss sheep population and the costs and benefits of Swiss-wide control programs. The joint analysis of the economic and epidemiological aspects of footrot allowed predicting the costs, benefits, and net economic effects under different control programs has not been implemented to date. By applying an epidemiological model, spatio-temporal prevalence information could be generated that served as basis for the economic analysis of the control strategies. Particularly in veterinary medicine, cost–benefit analysis is highly relevant for the decision whether or not to implement a disease control program.

The simulation model revealed that scenario C is most efficient in reducing nationwide footrot prevalence as fast as possible. This is due to the combination of the nationwide mandatory control program with the use of PCR diagnostics, which substantially increases the detection rate of infected animals. Nevertheless, this scenario was predicted to still require 6 and 10 years to reduce the Swiss-wide prevalence below 10 and 5%, respectively.

Global sensitivity analysis revealed that the recovery rate σ and reversion rate γ are most influential on the prediction of footrot prevalence over time. These parameters simulate the disease spread within the regions. Parameters defining the spread between regions, i.e., those related to common pasture, exhibitions, and sheep transport between regions, are less sensitive. This implies that in the current endemic footrot situation in Switzerland, the main effort should be directed toward reducing the prevalence within the regions. This finding is in line with experiences of the cantons GR and GL where the prevalence could be reduced significantly within a few years after the implementation of the mandatory control program (personal information cantonal veterinary office GR). However, reinfections have also been observed frequently through contact with infected animals on pastures or after purchase of infected sheep. Therefore, it can be hypothesized that the between region transmission pathways will become more relevant in an advanced control phase after the prevalence within the regions was successfully reduced. In this project, modification of between region pathways has not been investigated by restriction of sheep movements, pasturing, or participation at exhibitions. Yet, this is certainly worthwhile to be undertaken in the situation of advanced footrot management, because control measures restricted to regional activities are not sustainable enough.

From an economic point of view, it can be concluded that under current management costs and benefits, it is advisable to implement a systematic program that aims at a reduction of the footrot prevalence level. Over the long run management costs of individually tackling footrot are far higher than in a systematic, Swiss-wide approach, which is able to quickly reduce the prevalence of footrot in Switzerland. The analysis has shown that the net economic benefit increases with higher treatment intensity. Therefore, a systematic sanitation program with PCR method has been demonstrated to be the best choice.

An aspect is the potential economic effect of nationwide programs on the market price of sheep product markets. It has been demonstrated earlier that consumers are willing to pay higher prices for products yield from animal production with high welfare levels (37). For the Swiss sheep meat market, this effect is hard to predict and likely small due to several factors. On the one hand, a successful nationwide footrot program increases the number of healthy animals in Switzerland and with it the supply of sheep products. On the other hand, the high costs of implementing the mandatory measures might induce farmers to exit, which has adverse effects on the supply. The net effect of these diverging forces on the lamb meat market price is further dampened due to Swiss import regulations (potential adjustment of the import quota for lamb meat). The import quota is set quarterly by the Swiss meat association. A changing supply could, therefore, be compensated by higher or lower imports. However, it has to be noted that the minimum import amount set by the Uruguay round has to be 4,500 tons per year (38). During the last years, this threshold has always been exceeded, resulting in an import share of >50% of the Swiss sheep meat market (38).

Like all models, the simulation model is based on a series of assumptions. First, it was assumed that only one herd exists per premises. It might, therefore, be possible that the number of herds in Switzerland were underestimated. However, the influence on the output of the simulation model is expected to be negligible because the disease very likely spreads easily via pasturing or contaminated objects (e.g., foot-paring instruments) within the same premises even when more than one herd is kept. Second, neither disease transmission by migratory sheep flocks nor by cattle, goat, and wild ruminants were considered for the spread between regions. Migratory sheep flocks integrate sheep collected from different premises at the end of the pastoral season, and travel to the low land of Switzerland until they reach the weight to be slaughtered. Information on migration routes is not available in Switzerland. Therefore, uncertainties would have been too high to allow inclusion into the model. Also, only six migratory flocks are currently registered in Switzerland, their influence on the propagation of the disease is likely to be limited. The role of cattle in the transmission of virulent strains of *D. nodosus* leading to footrot in sheep is still under debate, although cross-infection between the two species in co-grazing settings was demonstrated (39, 40). In Switzerland, cattle and sheep are rarely kept in the same stable and are not transported together, which hampers potential transmission. It was demonstrated that the transmission of footrot is possible between goats and sheep when kept in close contact (41). However, in Switzerland sheep and goats are mainly kept together in smaller premises and hobby farms and the main part of sheep movements is caused by professional farmers. Therefore, it can be assumed that the role of goats in spread of footrot is negligible in Switzerland. Nevertheless, the influence of goats, but also other species such as wild ruminants on the spread of footrot to sheep should be further investigated.

In the presented work, epidemiological and economic models were combined to assess footrot management programs in the Swiss sheep population. It was found that a nationwide coordinated program with the use of the improved diagnostic test revealed to be the most cost-efficient strategy to control the disease. Implementation of such a program is therefore recommended from a scientific point of view.

## Ethics Statement

The animal experiment was approved by the Cantonal Veterinary Office of the Canton of Zug (approval number ZG 67/15) in accordance with the Swiss animal welfare legislation.

## Author Contributions

All authors were involved in the design of the study described here, with responsibilities in different parts. MA was another overall leader of this project. SD, DZ, GS, and GG were involved in the epidemiological part of this work. SS, MR, and CK were involved in the economical analysis. DZ, SD, SS, and CK were the main authors of this study. All co-authors read and commented the manuscript and approved the final version to be submitted.

## Conflict of Interest Statement

The authors declare that the research was conducted in the absence of any commercial or financial relationships that could be construed as a potential conflict of interest.

## Footnotes

^1^https://cran.r-project.org

^2^http://szv.caprovis.ch/

^3^http://bgk.caprovis.ch/cms05/showlinx.asp?lang=1&id=1

^4^The index for each farm was dropped in the NPV formula to simplify the notification.

^5^Focusing on the regression specification that replicates the data generating process most accurately; a detailed analysis of the different specifications can be found in the project report [Aepli et al. (25)].
