# Supplementary material for: Epidemiological and Economic Evaluation of Alternative On-Farm Management Scenarios for Ovine Footrot in Switzerland
Source: Front Vet Sci. 2017 May 16;4:70. doi: 10.3389/fvets.2017.00070 (PMC5432651; doi:10.3389/fvets.2017.00070)
Supplement: Supplementary file 1 [file presentation_1.pdf]

Appendix

1. Details on the compartmental transition rates

1.1 Infection rate β

The region specific infection rate β is a stochastic parameter (pert-distribution) which incorporates the regional sheep premises density and the climate. Climate is integrated via the climatic factor, which is calculated separately per region *i*.

$$Cl_i = \frac{\sum \bar{p}_i + \sum \bar{t}_i}{N_i} \quad (1)$$

The climatic factor (Cl_i) is the sum of the mean precipitation (p_i) and the mean temperature (t_i) per region *i* divided by the number of weather stations per region (N_i). Data of 80 weather stations was provided by MeteoSchweiz (www.meteoschweiz.admin.ch/home/service-und-publikationen/beratung-und-service/datenportal-fuer-lehre-und-forschung.html. Stations were selected using the criteria to having been working for the past 30 years and measuring the monthly precipitation as well as the mean monthly temperature 2 meters above the ground. Stations higher than 1750m a.s.l. (premises are rarely located higher than 1750m a.s.l.) and data from November to April were excluded because simulation were focused on the pasture season (Mai to October). The density factor d_i per region *i* was calculated as the number of premises holding sheep registered in the AGIS database per agricultural area of each region.

The factors Cl_i and d_i were successively related to the infection rate β of Rogaland (β_{Rog}), Norway, as described in Grøneng et al. (1), to relate the above defined climate and density factors to a real observed outbreak. As such, infection rate β was calculated as a pert-distribution (minimum, median, maximum) per region *i*:

$$\beta_i \sim Pert(\beta_{Min} = \beta_{Min,Rog} * \sqrt[2.3]{\frac{d_i}{d_{Rog}} * \frac{Cl_i}{Cl_{Rog}}},$$

$$\beta_{Mod} = \beta_{Mod,Rog} * \sqrt[2.3]{\frac{d_i}{d_{Rog}} * \frac{Cl_i}{Cl_{Rog}}},$$

$$\beta_{Max} = \beta_{Max,Rog} * \sqrt[2.3]{\frac{d_i}{d_{Rog}} * \frac{Cl_i}{Cl_{Rog}}}) , \quad (2)$$

where d_{Rog} (3.64 herds/km^2) is the herd density and Cl_{Rog} (standardized to 1) the climatic factor of Rogaland.

In a next step, β was set in the context of Switzerland by using information gained from the questionnaire study with sheep farmers. The proportion of farmers stated to have no problems with foot-rot in the years 2010-14 but then had the disease in 2014 out of the pool of farmers stated to have no problems with foot-rot in 2010-14 was defined as the infection rate *inf* and amounted to 9.5%. The final value of the infection rate β_{final} per region *i* was integrated in the model as follows:

$$\beta_{i,Mod\_final} = \frac{inf}{\sum_{i=1}^{27} \beta_{i,Mod} / 27} * \beta_{i,Mod} \quad (3)$$

$$\beta_{i,Min\_final} = \frac{\beta_{i,Min}}{\beta_{i,Mod}} * \beta_{i,Mod\_final} \quad (4)$$

$$\beta_{i,Max\_final} = \frac{\beta_{i,Max}}{\beta_{i,Mod}} * \beta_{i,Mod\_final} \quad (5)$$

The regional values of Cl_i, d_i and final β_i are presented in Table S1.

1.2 Recovery rate σ and reversion rate γ

The recovery rate and the reversion rate were calculated separately for premises that did and did not go through a foot-rot control program, respectively. This was done because these rates for these two premises groups were implemented with different values depending on the scenario and the region. The differentiation between the two premises groups was carried out with help of the question in the questionnaire interrogation whether control measure on herd level was executed or not.

The recovery rate σ was calculated out of the number of premises which had no problems with foot-rot in 2014 but did have foot-rot in 2010-14. The recovery rate was the percentage of these recovered premises out of the total number of infected premises in the years 2010-14. It was computed at 25.9% (entire questionnaire database), 45.6% (premises with herd level control measures undergone) and 22.3% (premises without herd level control measures applied).

The reversion rate γ was determined by the premises that had problems with foot-rot in 2010-14, carried out control measures with success (according to the farmer), but still had problems with foot-rot in 2014, which were interpreted to be re-occurring. Their percentage out of all foot-rot infected premises in 2010-14 is defined as γ. It was computed at 58.1% (entire questionnaire database), 43.6% (premises with herd level control measures undergone) and 74.5% (premises without herd level control measures applied).

An alternative reversion rate was calculated assuming that the detection of foot-rot was based on a PCR diagnostic test, increasing the sensitivity for disease detection. For this, the proportion of premises with foot-rot problem in 2014 out of those without any foot-rot problem in 2010-2014 (i.e. newly infected premises) was calculated. The calculated value was 9.5%.

All recovery and reversion rates were incorporated into the model as a uniform distribution with a variation of +/- 10% around the above calculated value.

2. Details on the disease spread pathways between regions

2.1 Sheep traded (θ)

The number of newly infected premises per year via sheep transports for trade between the regions was calculated for all possible combinations of sending regions *j* and receiving regions *i*.

$$\theta_{j,i} = \frac{I_j}{popSize_j} * MSh_{j,i} * ProbMove * \frac{S_i}{popSize_i}, \quad (6)$$

where $\frac{I_j}{popSize_j}$ denotes the proportion of infected premises in region *j*, $MSh_{j,i}$ denotes the number of sheep movement on herd level from region *j* to region *i* within a year and $\frac{I_j}{nSh_j} * MSh_{j,i}$ stands for the possibility that a herd from an infected premises is moved within a year. Regional population sizes ($popSize_j$ and $popSize_i$) are kept fixed over the period of the simulation (Table S1) and the numbers of susceptible and infected premises are changing with disease spread. *ProbMove* reflects the possibility that at least one animal per moved herd is infected and will thus transmit the disease into the new region. Uniform distribution was used for this parameter with a minimum of 0.01 (assuming within herd prevalence of 0.01) and a maximum of 0.995 (assuming a within herd prevalence of 0.65) according to Grøneng et al. (1). The proportion of susceptible premises in the receiving region *i* is denoted by $\frac{S_i}{popSize_i}$.

It was assumed that professional sheep farmers, who were integrated in the questionnaire dataset, caused the main part of sheep transports and that movements caused by non-professional farmers (not integrated in the questionnaire dataset) was negligible for the spread of foot-rot between regions. In the questionnaire, farmers were asked to state the two cantons – apart from the home canton – where the majority of sheep has been sent to or received from in the last year. The mean of all recorded acquisitions and sales was considered as the number of movements between each pair of regions. Each of these movements was counted as the movement of one herd independent of the number of sheep moved. The origin and destinations of the sheep transports were recorded on a cantonal level which does not agree with the regions in all cases. Therefore, it was assumed that the moved herds were evenly distributed over the sending and receiving cantons, respectively, and allocated to the respective regions. For example, if a herd was moved to canton A that is situated to one third in region 1 and to two thirds in region 2, then the probability that this herd will finally be sent to region 1 is half of the probability that it will be sent to region 2. Finally, the number of movements between regions was multiplied with the return rate of the questionnaire to extrapolate the number of sheep transports to entire Switzerland.

2.2 Common pasture (τ)

The number of newly infected premises via common pasture ($\tau_{j,i}$) per year was calculated using information on the yearly number of sheep herds from both region *i* and *j* that spend the summer on common pasture, herd density ($d_{pasture}$) and climate ($Cl_{pasture}$) on the pastures, the proportion of infected herds in region *j* ($\frac{I_j}{popSize_j}$) and the proportion of susceptible herds in region *i* ($\frac{S_i}{popSize_i}$). Regional population sizes are kept fixed over the period of the simulation (Table S1) and the numbers of susceptible and infected premises are changing with disease spread.

$$\tau_{j,i} = \beta_{pasture} * \frac{I_j}{popSize_j} * n_{pasture,j} * \frac{n_{pasture,i}}{n_{pasture,j}+n_{pasture,i}} * \frac{S_i}{popSize_i}, \quad (7)$$

where $n_{pasture,j}$ and $n_{pasture,i}$ denotes the number of herds from region *j* and *i* ,respectively, sent to common pasture. Region *i* is receiving and region *j* transmitting foot-rot infection. Herd density and climate on pasture are integrated in the transmission rate $\beta_{pasture}$, which is fitted to real outbreak data of Rogaland (Norway) as performed for the regional transmission rates β (equation 2).

$$\beta_{pastrue} \sim Pert(\beta_{Min} = \beta_{Min,Rog} * \sqrt[2.3]{\frac{d_{pastrue}}{d_{Rog}}} * \frac{Cl_{pasture}}{Cl_{Rog}},$$
$$\beta_{Mod} = \beta_{Mod,Rog} * \sqrt[2.3]{\frac{d_{pastrue}}{d_{Rog}}} * \frac{Cl_{pasture}}{Cl_{Rog}},$$
$$\beta_{Max} = \beta_{Max,Rog} * \sqrt[2.3]{\frac{d_{pastrue}}{d_{Rog}}} * \frac{Cl_{pasture}}{Cl_{Rog}}) \quad (8)$$

To calculate the herd density on pastures ($d_{pastrue}$), the recorded number of herds sent to pasture over summer time in the questionnaire study for those pasture areas which are registered in the AGIS database were divided by the pasture area size recorded in AGIS. The mean of the thereby calculated densities was defined as the herd density (2.39 herds/km^2) of all pasture areas in the model. The large majority (95%) of common pastures are situated on an altitude of 1000m a.s.l. and higher (according to http://www.srf.ch/sendungen/die-aelplerfamilie/definition-einer-alp). Therefore, only weather stations located >1000m a.s.l. were used for the calculation of the climate factor ($Cl_{pasture}$, 1.016) of the pasture areas, according to equation 1.

2.3 Sheep expositions (δ)

The number of newly infected premises per year via exhibitions ($\delta_{j,i}$) was calculated out of the number of sheep herds exhibited by region *i* ($n_{Expo,i}$) and *j* ($n_{Expo,j}$) per year, the herd density and climate on the site of exposition, the proportion of infected herds in region *j* ($\frac{I_j}{popSize_j}$) and the proportion of susceptible herds in region *i* ($\frac{S_i}{popSize_i}$), where region *i* is receiving and region *j* transmitting foot-rot infection. Regional population sizes are kept fixed over the period of the simulation (Table S1) and the numbers of susceptible and infected premises are changing with disease spread.

$$\delta_{j,i} = \beta_{Expo} * \frac{I_j}{popSize_j} * n_{Expo,j} * \frac{n_{Expo,i}}{n_{Expo,j}+n_{Expo,i}} * \frac{S_i}{popSize_i} \quad (9)$$

Herd density and climate on site of the exhibition are integrated in the transmission rate β_{Expo}, which is fitted to real outbreak data of Rogaland (Norway) as performed for the regional transmission rates β (equation 2).

$$\beta_{Expo} \sim Pert(\beta_{Min} = \beta_{Min,Rog} * \sqrt[2.3]{\frac{d_{Expo}}{d_{Rog}}} * \frac{Cl_{Expo}}{Cl_{Rog}},$$

$$\beta_{Mod} = \beta_{Mod,Rog} * \sqrt[2.3]{\frac{d_{Expo}}{d_{Rog}}} * \frac{Cl_{Expo}}{Cl_{Rog}},$$

$$\beta_{Max} = \beta_{Max,Rog} * \sqrt[2.3]{\frac{d_{Expo}}{d_{Rog}}} * \frac{Cl_{Expo}}{Cl_{Rog}}), \quad (10)$$

A list of all inter-cantonal sheep expositions (n=16) taken place in 2014 was provided by the Swiss Sheep Breeding Association. To determine the number of exhibited herds per region of origin, it was assumed that each exhibitor sent one herd, independent of the number of animals. The density of herds on an exposition per km^2 (d_{Expo}) was calculated based on the Swiss animal welfare regulation (https://www.admin.ch/opc/de/classified-compilation/20080796/index.html, Annex 1159, Table 4),

where a minimal area of at least $2m^2$ per animal was prescribes. This can be reduced in case of short-time husbandry (e.g. on expositions) and it was assumed that the density on expositions was 1 animal per m^2. As each exhibitor represented on average eight animals $8m^2$ were used for one herd, which resulted in a density of 125’000 herds per km^2 on expositions. The climate factor for the expositions (Cl_{Expo}; 0.121) was calculated according to equation 1, using information from weather stations located <1750m a.s.l. within the region where the expositions took place, and only considering precipitation and temperature during the month of exposition.

Reference

1. Grøneng GM, Vatn S, Kristoffersen A, Nafstad O, Hopp P. (2015) The potential spread of severe footrot in Norway if no elimination programme had been initiated: a simulation model. Vet Res 46:10
