# Supplementary material for: Epidemiological and Economic Evaluation of Alternative On-Farm Management Scenarios for Ovine Footrot in Switzerland
Source: Front Vet Sci. 2017 May 16;4:70. doi: 10.3389/fvets.2017.00070 (PMC5432651; doi:10.3389/fvets.2017.00070)
Supplement: Supplementary file 2 [file presentation_2.pdf]

Supplementary tables

Table S1: Input parameter of the model which were constant for all scenarios: Popsize = number sheep premises, Density = mean density of sheep premises per km^2, BetamaxCounty = maximum of the pert-distribution of the infection rate β per year, BetaminCounty = minimum of the pert-distribution of the infection rate β per year, BetamedCounty = median of the pert-distribution of the infection rate β per year, Climate factor = climatic factor, Infected = number of infected premises at the beginning of a simulation, Prevalence = outcome of the Maximum Entropy Model and starting prevalence of the epidemiological model

Region	PopSize	Density	Betamax County	Betamin County	Betamed County	Climate factor	Infected	Preva-lence
1	570	0.428	0.067	0.056	0.065	0.869	212	37.2%
2	184	0.251	0.059	0.049	0.057	0.970	68	37.2%
3	362	0.204	0.056	0.046	0.054	0.995	163	45.1%
4	110	0.348	0.050	0.042	0.049	0.713	50	45.1%
5	1027	0.315	0.058	0.048	0.056	0.857	371	36.1%
6	640	0.531	0.092	0.076	0.088	1.081	315	49.2%
7	2169	0.644	0.082	0.068	0.079	0.893	1064	49.1%
8	1052	0.611	0.073	0.061	0.071	0.812	423	40.2%
9	1027	0.872	0.089	0.074	0.086	0.847	368	35.8%
10	2074	1.186	0.138	0.114	0.132	1.143	784	37.8%
11	972	1.499	0.143	0.119	0.138	1.074	536	55.1%
12	967	3.988	0.116	0.096	0.111	0.567	208	21.5%
13	268	0.876	0.068	0.056	0.065	0.644	106	39.5%
14	37	0.504	0.052	0.043	0.050	0.625	15	39.5%
15	518	1.742	0.203	0.168	0.195	1.425	263	50.8%
16	1414	1.832	0.178	0.148	0.171	1.225	735	52.0%
17	949	0.784	0.105	0.087	0.101	1.041	374	39.4%
18	582	0.401	0.063	0.053	0.061	0.841	147	25.3%
19	91	0.408	0.056	0.047	0.054	0.743	23	25.3%
20	1105	0.654	0.089	0.074	0.085	0.953	469	42.5%
21	793	0.931	0.153	0.127	0.148	1.415	287	36.2%
22	1336	1.560	0.118	0.098	0.114	0.863	552	41.3%
23	124	0.886	0.139	0.115	0.134	1.311	36	28.8%
24	161	0.967	0.083	0.070	0.081	0.761	46	28.8%
25	223	1.187	0.114	0.094	0.109	0.944	33	14.7%
26	237	1.619	0.103	0.086	0.100	0.751	78	32.9%
27	492	1.549	0.126	0.105	0.121	0.933	171	34.7%

Table S2: Sheep population by region defined for the epidemiological model for 1999-2014

Region	Sheep population in 1999	Sheep population in 2014	Change (1999-2014)
1 + 2	7,561	11,413	50.9%
3 + 4	7,149	10,387	45.3%
5	12,029	15,618	29.8%
6	11,718	11,573	-1.2%
7	29,280	33,638	14.9%
8	16,150	17,125	6.0%
9	15,925	25,107	57.7%
10	19,609	24,968	27.3%
11	13,328	13,442	0.9%
12	36,578	31,543	-13.8%
13 + 14	14,375	18,747	30.4%
15	17,541	16,319	-7.0%
16	34,048	34,872	2.4%
17	13,282	18,693	40.7%
18 + 19	11,810	12,119	2.6%
20	20,874	28,849	38.2%
21	12,381	16,484	33.1%
22	32,622	31,373	-3.8%
23 + 24	9,765	9,944	1.8%
25	9,403	8,737	-7.1%
26	17,412	13,724	-21.2%
27	27,333	19,741	-27.8%
Total	390,173	424,416	8.8%

Table S3: Regression results for the Swiss sheep population predictions for 2014-2030 using the Seemingly Unrelated Regression model. Z-values provided in parenthesis. *** and ** indicate significance at 99% and 95% confidence level, respectively.

Region	α		β		R^2	(Chi^2)
1, 2*	6,896	(17.44)***	131.6	(3.22)***	0.39	(10.37)***
3, 4*	7,420	(10.97)***	-33.7	(-0.48)	0.01	(-0.23)
5	10,639	(16.13)***	318.0	(4.66)***	0.58	(21.74)***
6	10,155	(29.95)***	2.6	-0.08	0.00	(-0.01)
7	21,950	(19.92)***	575.0	(5.05)***	0.61	(25.47)***
8	13,601	(25.92)***	79.8	-1.47	0.12	(-2.16)
9	14,092	(16.68)***	705.6	(8.07)***	0.80	(65.2)***
10	17,074	(32.61)***	416.3	(7.69)***	0.79	(59.11)***
11	10,587	(20.48)***	117.3	(2.19)**	0.23	(4.81)**
12	36,284	(86.35)***	-418.6	(-9.63)***	0.85	(92.81)***
13, 14*	13,364	(23.40)***	129.9	(2.2)**	0.23	(4.83)**
15	18,266	(59.28)***	-216.5	(-6.79)***	0.74	(46.16)***
16	35,514	(53.16)***	37.3	-0.54	0.02	(-0.29)
17	13,346	(18.82)***	324.4	(4.42)***	0.55	(19.56)***
18, 19*	10,729	(31.32)***	113.6	(3.21)***	0.39	(10.28)***
20	22,045	(31.57)***	452.5	(6.27)***	0.71	(39.27)***
21	11,184	(28.76)***	283.2	(7.04)***	0.76	(49.57)***
22	34,647	(45.37)***	-120.5	(-1.53)	0.13	(-2.33)
23, 24*	9,821	(52.38)***	-44.5	(-2.29)**	0.25	(5.27)**
25	10,041	(47.02)***	-65.3	(-2.96)***	0.35	(8.74)***
26	17,632	(82.55)***	-299.6	(-13.57)***	0.92	(184.01)***
27	27,726	(101.02)***	-561.0	(-19.77)***	0.96	(390.67)***

*these regions were compiled for the regression analysis

Table S4: Farm size defined by number of sheep per farm (number of farms, and percentage in brackets) by region in Switzerland in 2014.

Region	Small (1-30)	Medium (31-70)	Large (>70)
1 + 2	391 (83.73%)	45 (9.64%)	31 (6.64%)
3 + 4	230 (80.14%)	30 (10.45%)	27 (9.41%)
5	504 (82.49%)	60 (9.82%)	47 (7.69%)
6	318 (78.33%)	58 (14.29%)	30 (7.39%)
7	1076 (82.14%)	145 (11.07%)	89 (6.79%)
8	532 (78.35%)	99 (14.58%)	48 (7.07%)
9	609 (74.27%)	128 (15.61%)	83 (10.12%)
10	1194 (87.22%)	120 (8.77%)	55 (4.02%)
11	415 (78.75%)	76 (14.42%)	36 (6.83%)
12	192 (31.95%)	293 (48.75%)	116 (19.3%)
13 + 14	94 (48.45%)	38 (19.59%)	62 (31.96%)
15	164 (51.9%)	76 (24.05%)	76 (24.05%)
16	514 (58.54%)	235 (26.77%)	129 (14.69%)
17	526 (78.51%)	77 (11.49%)	67 (10%)
18 + 19	375 (78.29%)	69 (14.41%)	35 (7.31%)
20	472 (69.82%)	114 (16.86%)	90 (13.31%)
21	451 (75.42%)	97 (16.22%)	50 (8.36%)
22	543 (65.58%)	176 (21.26%)	109 (13.16%)
23 + 24	109 (56.48%)	44 (22.8%)	40 (20.73%)
25	87 (50.29%)	47 (27.17%)	39 (22.54%)
26	46 (26.59%)	55 (31.79%)	72 (41.62%)
27	146 (42.2%)	117 (33.82%)	83 (23.99%)
Total	8988 (71.33%)	2199 (17.45%)	1414 (11.22%)

Table S5: Predictions of future sheep farm population in Switzerland (2014 – 2030) using the Seemingly Unrelated Regression model. The values are yearly regression coefficients *β* using large farms (<70 animals) as reference level. Z-values provided in parenthesis. *** indicate significance at 99% confidence level.

Variable	Small (1-30 sheep/farm)		Medium (31-70 sheep/farm)	
Trend	-0.0012	(-4.2)***	-0.0017	(-7.14)***
Region 1, 2[$]	0.8514	(128.85)***	0.1204	(21.83)***
Region 3, 4[$]	0.7550	(114.27)***	0.1293	(23.44)***
Region 5	0.8336	(126.16)***	0.1263	(22.89)***
Region 6	0.8013	(121.27)***	0.1582	(28.67)***
Region 7	0.8496	(128.58)***	0.1231	(22.32)***
Region 8	0.8179	(123.79)***	0.1498	(27.15)***
Region 9	0.7768	(117.57)***	0.1739	(31.53)***
Region 10	0.9011	(136.38)***	0.0947	(17.17)***
Region 11	0.7990	(120.92)***	0.1689	(30.62)***
Region 12	0.3920	(59.33)***	0.5012	(90.86)***
Region 13, 14[$]	0.5020	(75.98)***	0.2453	(44.46)***
Region 15	0.4113	(62.25)***	0.3791	(68.71)***
Region 16	0.6351	(96.12)***	0.2728	(49.45)***
Region 17	0.8188	(123.92)***	0.1274	(23.09)***
Region 18, 19[$]	0.7632	(115.51)***	0.1680	(30.46)***
Region 20	0.7592	(114.9)***	0.1670	(30.27)***
Region 21	0.8057	(121.94)***	0.1648	(29.88)***
Region 22	0.6714	(101.62)***	0.2345	(42.51)***
Region 23, 24[$]	0.5859	(88.68)***	0.2862	(51.88)***
Region 25	0.5320	(80.51)***	0.3005	(54.46)***
Region 26	0.3106	(47.01)***	0.3669	(66.51)***
Region 27	0.4662	(70.56)***	0.3745	(67.89)***
R^2 (Chi²)	0.9987	(280889.78)***	0.9922	(44586.29)***

[$]these regions were compiled for the regression analysis

Table S6: Prevalence of foot-rot for the four simulated scenarios A-D over the course of the simulation (57 years) [xls document]

The prevalence in region 24, 26 and 27 increased > 100% for scenario D. This was caused by the correction algorithm described in section 2.5 of the main text. The prevalence estimations of for these regions and time periods with values > 100% are not further usable. However, the correction algorithm does not influence the predictions of the national prevalence.
