# Supplementary material for: Epidemiological and Economic Evaluation of Alternative On-Farm Management Scenarios for Ovine Footrot in Switzerland
Source: Front Vet Sci. 2017 May 16;4:70. doi: 10.3389/fvets.2017.00070 (PMC5432651; doi:10.3389/fvets.2017.00070)

Figure S1: Swiss sheep population for 1999-2030. Data before 2014 are based on the farm accounting database and data after 2014 represents the linear prediction

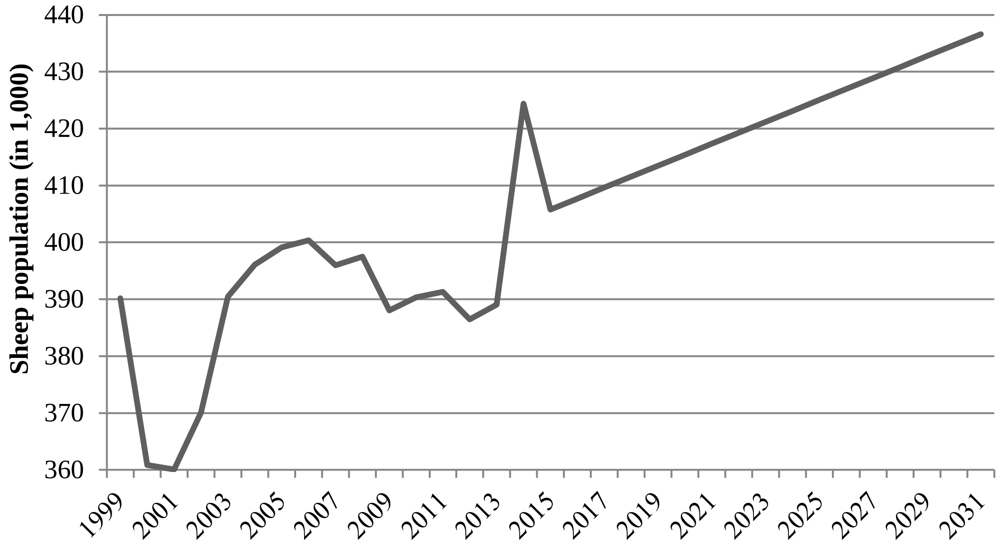

Figure S2: Relative frequencies of the three compartments during the fitting process. Blue = susceptible (S), green = infected (I), red = recovered (R). The thick black line reflects the year 45, the thin black line marks the point of intersection of the vertical line and the median of the I compartment (40.38%).

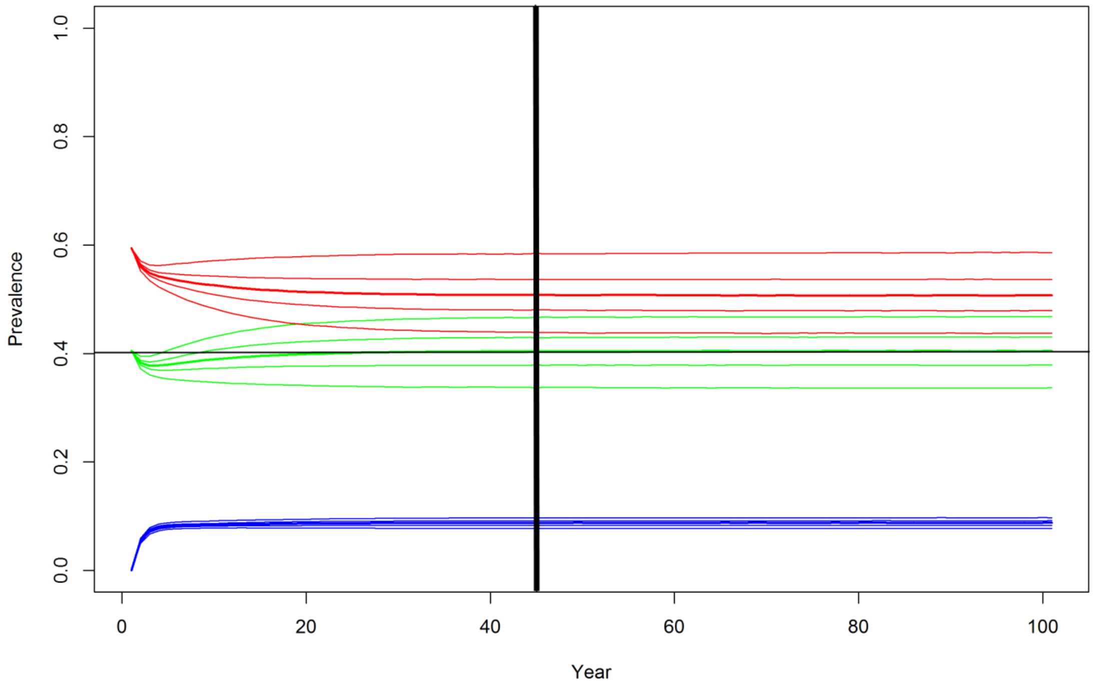

Figure S3: Output of the simulation model from the start of the simulation until year 100 (end of the simulation). Relative frequency of the susceptible (S, red), infected (I, green) and recovered (R, blue) population are illustrated using scenario C as an example. Scenario specific parameter values were implemented at year 45, which reflects the year 2014 in real time. For further analyses only the results after year 45 were considered. The lines for each compartment (S, I and R) represent the 2.5, 25, 50, 75 and 97.5 percentile.

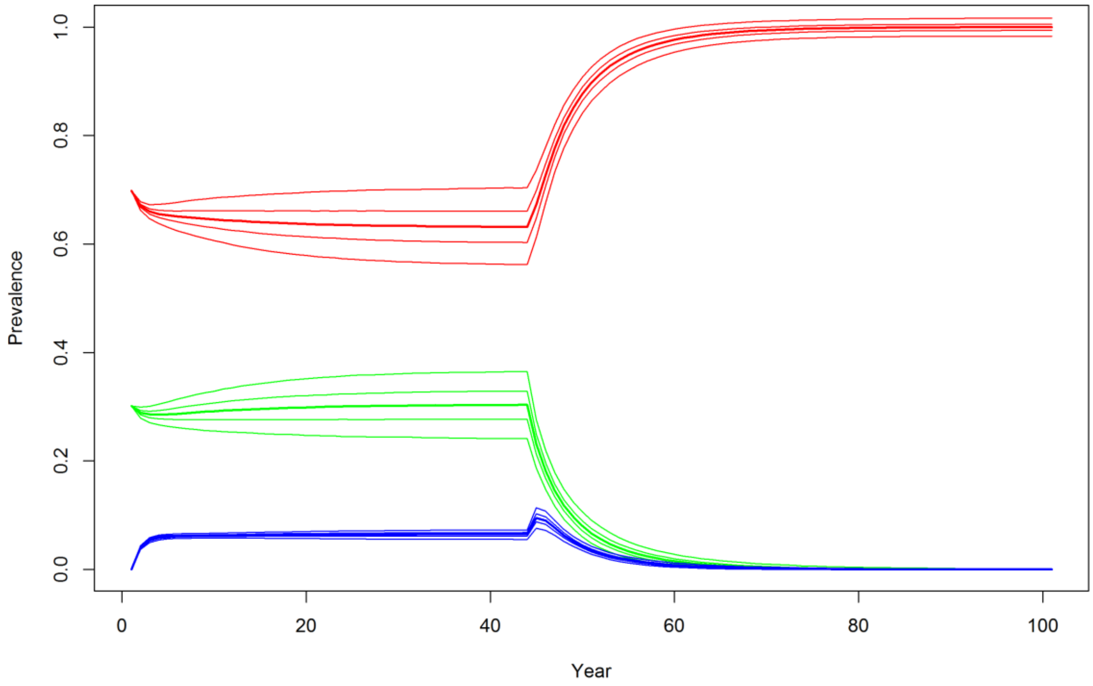

Figure S4: Sobol indices of the 13 parameters included into the global sensitivity analysis. The higher the index, the larger the influence on the model's outcome (foot-rot prevalence) is. The main effect index considers only the variation of the respective parameter while all other parameter values remained in the default value. The total effect index integrated the interaction between the respective parameter with all other parameters and is therefore considered to be more informative. Parameter values are: a=$Cl_{pasture}$ (climate factor on common pastures), b=Cl_{Expo} (climate factor on exhibitions), c= mean of all regional infection rates β_i, d= $d_{pasture}$ (herd density on common pastures), e= $n_{pasture}$ (number of sheep herds sent to common pastures), f= d_{Expo} (herd density on exhibitions), g= n_{Expo} (number of sheep herds sent to exhibitions), h= MSh_{ij} (number of sheep herd transports between region *i* and *j*), i= S_i (number of susceptible herds at the start of the simulation), j= I_i (number of infected herds at the start of the simulation), k= β_i (infection rate), l= σ_i (recovery rate), m= γ_i (reversion rate)

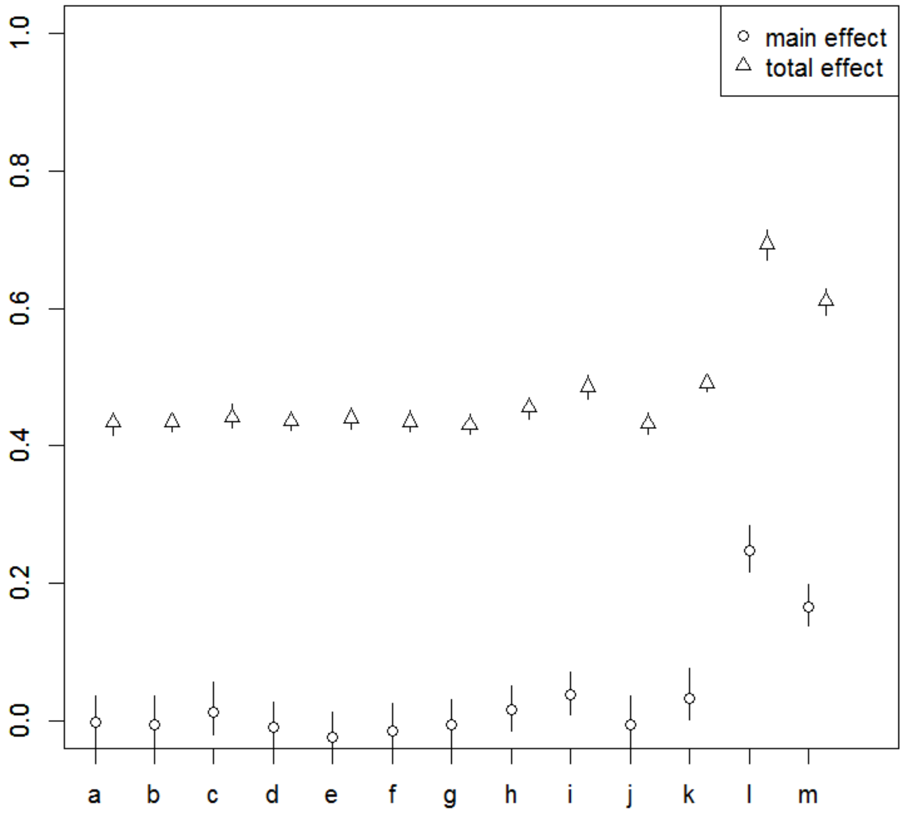

Supplement: Supplementary file 3 [file presentation_3.pdf]
